# Fault Identification of Chemical Processes Based on k-NN Variable Contribution and CNN Data Reconstruction Methods

**DOI:** 10.3390/s19040929

**Published:** 2019-02-22

**Authors:** Guo-Zhu Wang, Jing Li, Yong-Tao Hu, Yuan Li, Zhi-Yong Du

**Affiliations:** 1 Department of Automatic Control, Henan Institute of Technology, Henan 453003, China; lijing2016@hait.edu.cn (J.L.); hythait@163.com (Y.-T.H.); zhydu@126.com (Z.-Y.D.); 2 Information Engineering School, Shenyang University of Chemical Technology, Liaoning 110142, China; li-yuan@mail.tsinghua.edu.cn

**Keywords:** fault detection, fault identification, k-nearest neighbor, center-based nearest neighbor, data reconstruction

## Abstract

Data-driven fault detection and identification methods are important in large-scale chemical processes. However, some traditional methods often fail to show superior performance owing to the self-limitations and the characteristics of process data, such as nonlinearity, non-Gaussian distribution, and multi-operating mode. To cope with these issues, the k-NN (k-Nearest Neighbor) fault detection method and extensions have been developed in recent years. Nevertheless, these methods are primarily used for fault detection, and few papers can be found that examine fault identification. In this paper, in order to extract effective fault information, the relationship between various faults and abnormal variables is studied, and an accurate “fault–symptom” table is presented. Then, a novel fault identification method based on k-NN variable contribution and CNN data reconstruction theories is proposed. When there is an abnormality, a variable contribution plot method based on k-NN is used to calculate the contribution index of each variable, and the feasibility of this method is verified by contribution decomposition theory, which includes a feasibility analysis of a single abnormal variable and multiple abnormal variables. Furthermore, to identify all the faulty variables, a CNN (Center-based Nearest Neighbor) data reconstruction method is proposed; the variables that have the larger contribution indices can be reconstructed using the CNN reconstruction method in turn. The proposed search strategy can guarantee that all faulty variables are found in each sample. The reliability and validity of the proposed method are verified by a numerical example and the Continuous Stirred Tank Reactor system.

## 1. Introduction

In modern chemical processes, fault detection and identification have become important tasks to ensure process safety, product quality, and environmental sustainability [1,2,3]. The effective detection and identification of abnormal operations are also crucial concerns for engineers and operators [1,4,5,6]. Many fault detection and identification methods have been proposed and detailed in several studies [2,3,4,5,6,7,8]. Moreover, due to the development of sensors, computer applications, and distributed control technology, extensive chemical process data can be collected and stored [9,10], so data-driven methods reveal many advantages [11,12]. Compared with the methods based on knowledge and analytical models, data-driven methods that are implemented only by analyzing and mining useful information for fault detection and diagnosis do not require precise mathematical modeling and process knowledge [13,14,15]. For example, principal component analysis (PCA) can extract the principal components (PCs) that effectively represent almost all information in the training data set, and the statistics Hotelling’s $T^{2}$ and squared prediction error (SPE) are constructed for fault detection [16]. Recently, some experts and scholars proposed some variations of data-driven methods to obtain better detection performances [17,18,19,20,21,22,23]. Fault detection methods based on k-Nearest Neighbor (k-NN) theory have been developed to successfully monitor continuous and batch processes [20,24]. However, due to the large amount of calculation involved in these methods and the need to store many intermediate values, the k-NN method has higher requirements for calculation speed and computer storage space. In order to solve these problems, He et al. proposed a PC-k-NN method, which uses the principal component of the original sample as a modeling sample to reduce the computational effort and save storage space [25]. However, the method ignores anomalous information that occurs in the residual space. Subsequently, Li et al. proposed a batch process fault detection method that was based on Feature Space k-Nearest Neighbor (FS-k-NN), which combines the principal part and the residual part of the feature space, and it achieved satisfactory results [26].

As is well known, fault detection is very important for ensuring the safety of chemical processes. Although the fault detection method based on k-NN theory has achieved satisfactory results, there are few studies on fault diagnosis and abnormal variable identification. Once abnormality has been detected, it is necessary to extract effective fault information, study the relationship between various faults and abnormal variables, establish an accurate “fault–symptom” table, and, finally, search for the fault roots from a set of possible faulty variables. So, fault identification is the next significant task. In the framework of statistical theory, a contribution plot is commonly used to identify anomalous variables. Such plots can be divided into an SPE contribution plot and $T^{2}$ contribution plot [27]. These methods can be used to visualize the contribution value of each variable in the form of a histogram, so the variables that contribute the most to the statistical indicators are considered to be the responsible variables that may cause a process failure. However, the methods do not determine the control threshold of the contribution variables: that is, the contribution of each variable only plays a guiding role, and the ultimate cause of a fault needs further analysis and determination. In other words, they can only sort the variables according to their contributions and then roughly locate the abnormal variables, but the current methods cannot identify all abnormal variables in the process [28]. Kourti and MacGregor used this method to find faulty quality-related variables and process variables of a high-pressure low-density polyethylene reactor, and they also suggested that contribution plots may not always reveal assignable causes of abnormal events [29]. In addition, a reconstruction-based approach was proposed for isolating faulty variables from the subspace of faults [30], Carlos and Qin also proposed a reconstruction-based contribution for process monitoring and fault diagnosis [31]. These methods have been applied to reconstruct the data of faulty variables before performing a prediction for a soft sensor model. A combined index of SPE and $T^{2}$ was developed to isolate faulty variables [32], and it achieved more feasible solutions than the reconstruction-based approach. This is because the reconstruction-based contribution (RBC) approach does not suffer from the smearing effect that afflicts the contribution plots of PCA. In fact, the confidence intervals of RBC plots and the control limits were derived on the basis of normal operating data, and the magnitude of the smearing of faulty variables compared with the non-faulty ones was under the corresponding control limits, so the smearing effect of RBC can be observed [33]. Thus, fault identification for chemical processes on the basis of k-NN variable contribution and CNN data reconstruction methods has certain challenges, and it also has certain academic research value and practical significance.

This paper proposes a novel fault identification method based on k-NN variable contribution and CNN data reconstruction methods for chemical processes. First, the k-NN strategy is applied to normal process modeling and fault detection to check whether there are abnormalities in a real-time process. Second, when there is an abnormality detected in a real-time sample, a variable contribution plot method based on k-NN is proposed; it is similar to the classical contribution plot used for calculating the contribution index of each variable, and the feasibility of this method is verified by contribution decomposition theory, which includes a feasibility analysis of a single abnormal variable and multiple abnormal variables. Third, the variables that have larger contribution indices can be reconstructed using the CNN reconstruction method. Fourth, for the new sample (after reconstruction), the first step is revisited to check whether there are abnormalities. Finally, all faulty variables are determined. The method proposed in this paper has the following advantages: (1) the k-NN method can deal with non-Gaussian and nonlinear characteristics of modeling data effectively from the aspect of fault detection. (2) The CNN method can ensure accuracy of data reconstruction. Furthermore, this method can also ensure the effectiveness of fault identification.

The rest of this paper is organized as follows: we start by recalling the main idea of the k-nearest neighbor method and k-NN fault detection framework in Section 2. The proposed fault detection and identification methods are described in Section 3, including the k-NN contribution method and the feasibility of this method. The data reconstruction strategy and faulty variable identification using the CNN method are proposed and described in Section 4. In Section 5, a numerical example and the Continuous Stirred Tank Reactor system are introduced to illustrate the effectiveness of the proposed method from the aspect of fault detection and identification. The concluding remarks and plans for future work are provided in Section 6.

## 2. Preliminaries

### 2.1. The Rule of k-Nearest Neighbor

Recently, k-NN theory has been used for data classification, process monitoring, fault detection, image processing, and other fields as a nonparametric supervised classification method, and it has unique advantages in these applications [25,26]. For data classification, similar attributes among samples are divided in the learning sample using the k-NN method. The unknown class or label of an observation can then be predicted. The basic idea of the k-NN method is that if an observation point is close enough to a class with almost all the same samples, it should have the same attributes as this type of data; otherwise, it is different from these samples. Details of the k-NN method are as follows.

Consider a training data set $X_{n\times m}$, where *n* is the number of samples, and *m* is the number of variables. The *i*th sample is $x_{i}=\left[v_{1},v_{2},\ldots ,v_{m}\right]$, $v_{m}$ is the *m*th variable in each sample, and all of the sample points belong to the same class and have the same properties. There are several important concepts that must be explained to describe the k-NN classification method better:

(1) *k* represents the number of selected neighbor samples, so it is a positive integer fixed by the experimenter;

(2) $n_{k}\left(x_{i}\right)$ is the *k*th nearest neighbor of $x_{i}$.

Assuming there is a new sample $x_{new}$, the statistical index $D_{k}^{2}\left(x_{new}\right)$ can be calculated according to Equation (1). $x_{new}$ belongs to the same class as *X* when the cumulative distance index $D_{k}^{2}\left(x_{new}\right)$ is small enough. Conversely, $x_{new}$ is different from other samples in *X*.

(1) $$D_{k}^{2}\left(x_{new}\right)=\sum_{j=1}^{k}{∥x_{new}-n_{j}\left(x_{new}\right)∥}^{2}.$$

### 2.2. Fault Detection Method Using k-NN Theory

Because the k-NN rule has certain advantages in the field of data classification, it has been widely applied for pattern classification and fault detection. A fault detection method based on the k-nearest neighbors (FD-k-NN) rule [24] and its extension were researched to answer some specific questions, such as the analysis of non-Gaussian, nonlinear data and the computation of complex calculations. When the data reveal non-Gaussian statistics, the monitoring results based on the PCA method may lead to false alarms and produce undesired results. Unlike PCA, FD-k-NN constructs the threshold using the kernel density estimation (KDE) method [34], so the k-NN method can effectively deal with non-Gaussian data. In addition, it makes no assumption of the linearity of the data set because the k-NN rule is a nonlinear classifier.

**Figure 1:**
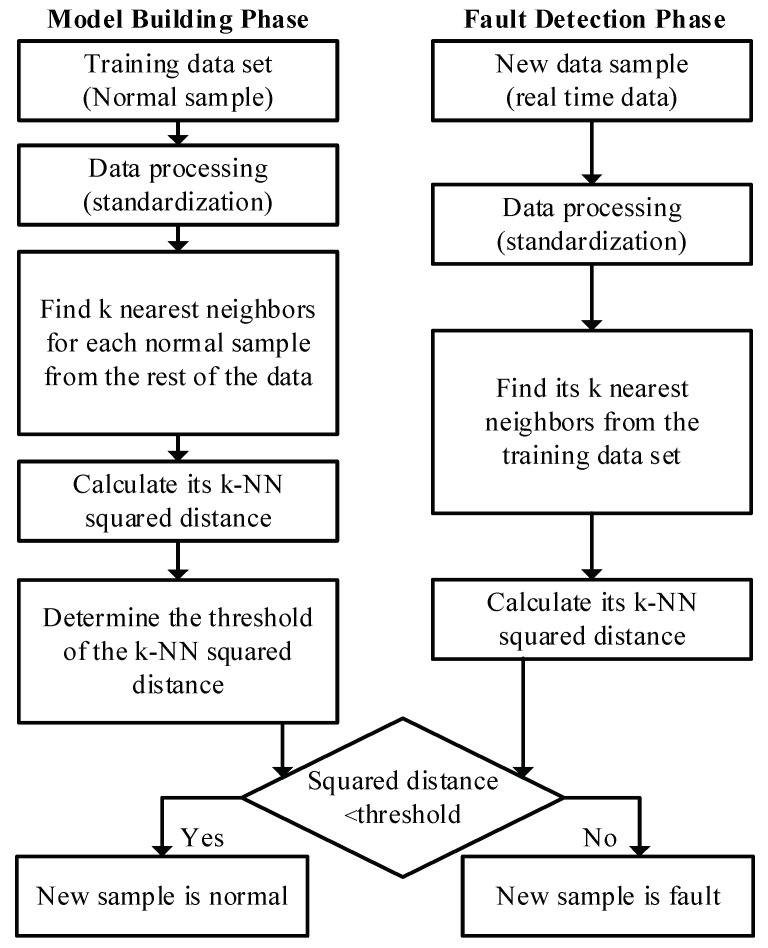
Flowchart of the FD-k-NN method.

From the perspective of fault detection, there is only one class of data (normal operating data) available as training data, as the basic idea of FD-k-NN is that the trajectory of a new normal sample is similar to the training samples; on the other hand, the trajectory of a new faulty sample must show some deviation from normal training samples. In other words, relative to a given threshold, the distance between a faulty sample and the nearest neighboring training samples must be greater, and the distance between a normal sample and the nearest neighboring training samples must be smaller. However, the training samples only contain normal samples, and there are no faulty samples under normal operating conditions. Therefore, if we can determine the distribution of the distances between a training sample and its nearest neighboring training samples, a threshold distance for a given confidence level will be defined. When the new samples’ distance to its nearest neighboring training samples is below the threshold, it is considered normal. Otherwise, it is a fault. The FD-k-NN method is described in detail below, which consists of two parts: model building and fault detection phases, as shown in Figure 1.

Model building phase:

(1) Collect and standardize the training data: the z-score data standardization method is used to scale each variable to the same level—i.e., for a given variable, the value of each sample in the training data minus the mean and divided by the standard deviation of the variable.

(2) For each standardized sample, find its *k* nearest neighbors in the training data set using the Euclidean distance as the indicator. For example, the *i*th neighbor of $x_{1}$ called $n_{i}\left(x_{1}\right)$.

(3) Calculate the k-NN squared distance for each sample: $D_{i}^{2}$ is the sum of squared distances of sample $x_{i}$ to its k-nearest neighbors, as calculated according to Equation (2),

(2) $$D_{k}^{2}\left(x_{i}\right)=\sum_{j=1}^{k}{∥x_{i}-n_{j}\left(x_{i}\right)∥}^{2}.$$

(4) Determine the 95% or 99% confidence limit $D_{\alpha }^{2}$ using the KDE method [34]. 

Fault detection phase: 

(1) For a new unknown sample $x_{new}$, standardize it using the mean and variance of the training data and find its *k* nearest neighbors in the training data set;

(2) Calculate its k-NN squared distance $D_{x_{new}}^{2}$;

(3) Compare $D_{x_{new}}^{2}$ with the threshold $D_{\alpha }^{2}$. If $D_{x_{new}}^{2}<D_{\alpha }^{2}$, it is a normal sample. Otherwise, it is detected as a fault.

## 3. The k-NN Variable Contribution Theory

The k-NN contribution analysis method is similar to the traditional contribution plot, and it can give the contribution value of all variables to a control index. When there is a fault, the k-NN distance contribution values of each variable can be calculated and compared. According to the k-NN modeling process, the k-NN squared distance $D_{k}^{2}\left(x_{i}\right)$ can be transformed into Equation (3):

(3) $$D_{k}^{2}\left(x_{i}\right)=\sum_{l=1}^{m}\sum_{j=1}^{k}{\left\{\left[x_{i}-N_{j}\left(x_{i}\right)\right]\varepsilon_{l}^{T}\right\}}^{2},$$

where $\varepsilon_{l}$ is a row vector, its *l*th element is 1, and the remaining elements are 0.

Here, the contribution value of the *l*th variable in the sample $x_{i}$ to the distance statistic index $D_{k}^{2}\left(x_{i}\right)$ can be defined as Equation (4),

(4) $$C_{il}=\sum_{j=1}^{k}{\left\{\left[x_{i}-N_{j}\left(x_{i}\right)\right]\varepsilon_{l}^{T}\right\}}^{2}.$$

According to Equations (3) and (4), the relationship between the k-NN statistical index and the distance contribution value of each variable is as follows:

(5) $$D_{k}^{2}\left(x_{i}\right)=\sum_{l=1}^{m}C_{il}.$$

According to the above analysis, the contribution value of the *l*th variable of sample $x_{i}$ to the distance statistical index is actually equal to the *l*th component of the square distance between the sample and its *k*th nearest neighbor. That is to say, the influence of the variable itself is only considered in this method, and the relationship between variables is not considered, which can effectively avoid smearing or diffusion effects between variables. However, when the process is faulty, the contribution of abnormal variables to statistical indicators is greater than that of other variables. This needs to be further explained. The feasibility of the k-NN variable contribution method is analyzed for two cases: a single abnormal variable and multiple abnormal variables.

### 3.1. The Feasibility Analysis of Single Abnormal Variable

Assume that $x_{f}$ is a faulty sample, and the *r*th variable deviates from the normal operating range. $x_{f}$ can be broken down into the following forms

(6) $$\begin{array}{c} x_{f}=x^{*}+\varepsilon_{r}f_{r}, \end{array}$$

where $x^{*}$ is the normal component of $x_{f}$, and $\varepsilon_{r}f_{r}$ is the faulty component. $\varepsilon_{r}$ is the direction of the fault, and $f_{r}$ is the amplitude of the fault in the corresponding direction. According to Equations (4) and (6), the contribution value of each variable can be obtained by Equation (7):

(7) $$\begin{array}{c} \begin{array}{c} C_{fl}=\sum_{j=1}^{k}{\left\{\left[x_{f}-N_{j}\left(x_{f}\right)\right]\varepsilon_{l}^{T}\right\}}^{2}=\sum_{j=1}^{k}{\left\{x^{*}+\varepsilon_{r}f_{r}-N_{j}\left(x_{f}\right)]\varepsilon_{l}^{T}\right\}}^{2}, \end{array} \end{array}$$

where $N_{j}\left(x_{f}\right)$ is the *j*th nearest neighbor of $x_{f}$ in the training data set, and $x^{*}$ is the normal component of $x_{f}$, so the following relations exist: $x^{*}-N_{j}\left(x_{f}\right)\approx 0$. The contribution value of each variable can be transformed into Equation (8):

(8) $$\begin{array}{c} \begin{array}{c} C_{fl}\approx \sum_{j=1}^{k}{\left\{\left[\varepsilon_{r}f_{r}\right]\varepsilon_{l}^{T}\right\}}^{2}=k{\left(\varepsilon_{r}f_{r}\varepsilon_{l}^{T}\right)}^{2}, \end{array} \end{array}$$

where $C_{fl}$ is the contribution value of the *l*th variable of sample $x_{f}$ to the distance statistical index, and $\varepsilon_{r}$ and $\varepsilon_{l}$ should satisfy Equation (9):

(9) $$\begin{array}{c} \varepsilon_{r}\varepsilon_{l}^{T}=\left\{\begin{array}{c} 0,r\neq l,\\ 1,r=l. \end{array}\right. \end{array}$$

From the above, when *r* is not equal to *l*, r≠l, the greatest contribution of abnormal variables is $C_{fr}$

(10) $$\begin{array}{c} C_{fr}=kf_{r}^{2}>C_{fl}\approx 0. \end{array}$$

The following conclusion can be drawn according to the above analysis: The k-NN distance contribution analysis method can ensure that an abnormal variable has the greatest contribution value when only a single variable is abnormal.

### 3.2. The Feasibility Analysis of Multiple Abnormal Variables

Assume that $x_{f}$ is a faulty sample and that several variables deviate from the normal operating range: variable *a*, variable *b*, and variable *c*. $x_{f}$ can be broken down into the following forms:

(11) $$\begin{array}{c} x_{f}=x^{*}+\varepsilon_{a}f_{a}+\varepsilon_{b}f_{b}+\varepsilon_{c}f_{c}, \end{array}$$

where $x^{*}$ is the normal component of $x_{f}$, $\varepsilon_{a}f_{a}$, $\varepsilon_{b}f_{b}$, and $\varepsilon_{c}f_{c}$ are the faulty components. ε and *f* are the directions and amplitudes of the fault. According to Equations (4) and (11), the contribution value of each variable can be obtained as Equation (12):

(12) $$\begin{array}{c} \begin{array}{c} C_{fl}=\sum_{j=1}^{k}{\left\{\left[x_{f}-N_{j}\left(x_{f}\right)\right]\varepsilon_{l}^{T}\right\}}^{2}=\sum_{j=1}^{k}{\left\{x^{*}+\varepsilon_{a}f_{a}+\varepsilon_{b}f_{b}+\varepsilon_{c}f_{c}-N_{j}\left(x_{f}\right)]\varepsilon_{l}^{T}\right\}}^{2}. \end{array} \end{array}$$

The following relations exist: $x^{*}-N_{j}\left(x_{f}\right)\approx 0$. The contribution value of each variable can be transformed into Equation (13):

(13) $$\begin{array}{c} \begin{array}{c} C_{fl}\approx \sum_{j=1}^{k}{\left\{\left[\varepsilon_{a}f_{a}+\varepsilon_{b}f_{b}+\varepsilon_{c}f_{c}\right]\varepsilon_{l}^{T}\right\}}^{2}=k{\left(\varepsilon_{a}f_{a}\varepsilon_{l}^{T}+\varepsilon_{b}f_{b}\varepsilon_{l}^{T}+\varepsilon_{c}f_{c}\varepsilon_{l}^{T}\right)}^{2}. \end{array} \end{array}$$

Similar to a single abnormal variable, when a,b,c≠l, $C_{fa}$, $C_{fb}$ and $C_{fc}$ can be represented as Equation (14):

(14) $$\begin{array}{c} \left\{\begin{array}{c} C_{fa}=kf_{a}^{2}>C_{fl}\approx 0,\\ C_{fb}=kf_{b}^{2}>C_{fl}\approx 0,\\ C_{fc}=kf_{c}^{2}>C_{fl}\approx 0. \end{array}\right. \end{array}$$

The following conclusion can be drawn according to the above analysis: The k-NN distance contribution analysis method can ensure abnormal variables have the greater contribution value when there are several abnormal variables.

## 4. Data Reconstruction and Faulty Variable Identification Strategy

In order to solve the multi-sensor fault problem, the k-NN and proposed CNN data reconstruction strategy are described in detail below, and the comparison and analysis of two methods are given in Section 4.3.

### 4.1. The k-NN Data Reconstruction Method

In this subsection, the data reconstruction method based on k-NN is given [35]. When there is a fault in a new sample, we can use the data reconstruction method as follows.

Step 1: Standardize the new sample using the mean and variance of the training data. The result is $x_{new}$, and $x_{new}=\left[v_{1},v_{2},\ldots ,v_{m}\right]$, $v_{i}$ is the label of the variable.

**Figure 2:**
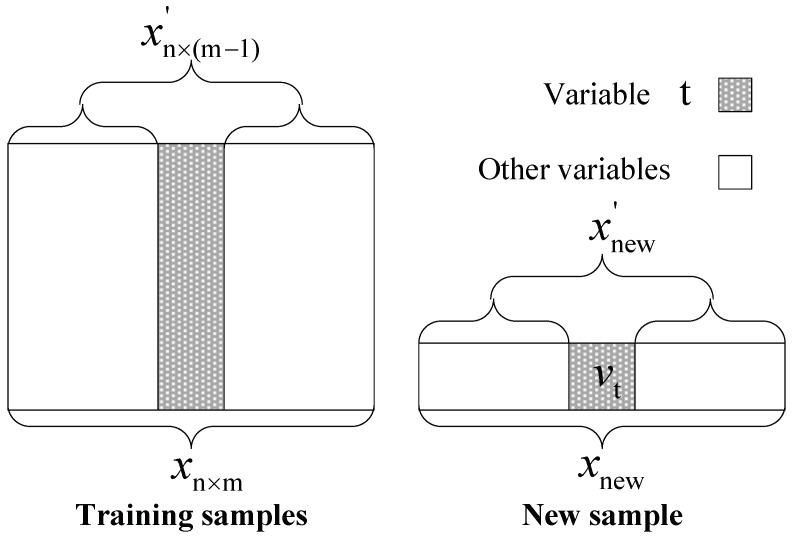
Flowchart of variable restructuring procedure.

Step 2: Reconstruct each variable $v_{1},v_{2},\ldots ,v_{m}$ according to Equation (15) and Figure 2, where $n_{i}\left(x_{new}^{{}^{\prime }}\right)$ is the *i*th nearest neighbor of $x_{new}^{{}^{\prime }}$ in training sample $x_{n\times (m-1)}^{{}^{\prime }}$, and ${\left[x_{n_{i}\left(x_{new}^{{}^{\prime }}\right)}\right]}_{t}$ is the *t*th variable of $x_{new}^{{}^{\prime }}$. The parameter *k* is a constant value of experience.

(15) $$v_{t}^{{}^{\prime }}=\frac{1}{k}\sum_{i=1}^{k}{\left[x_{n_{i}\left(x_{new}^{{}^{\prime }}\right)}\right]}_{t}.$$

### 4.2. The CNN Data Reconstruction Method

In this section, the CNN data reconstruction strategy is proposed. When there is a fault in the new sample, we can use the CNN data reconstruction method as follows:

Step 1: Standardize the new sample using the mean and variance of the training data, the result is $x_{new}$, and $x_{new}=\left[v_{1},v_{2},\ldots ,v_{m}\right]$, $v_{i}$ is the label of the variable.

Step 2: Determine the *k* value, which should satisfy Equation (16) according to Figure 2:

(16) $$f\left(k\right)=min[x_{new}^{{}^{\prime }}-\frac{1}{k}\sum_{i=1}^{k}n_{i}\left(x_{new}^{{}^{\prime }}\right)].$$

Step 3: Reconstruct the *t*th variable of $x_{new}$ according to Equation (15) when the *k* value is determined.

### 4.3. Comparison and Analysis of Two Methods

**Figure 3:**
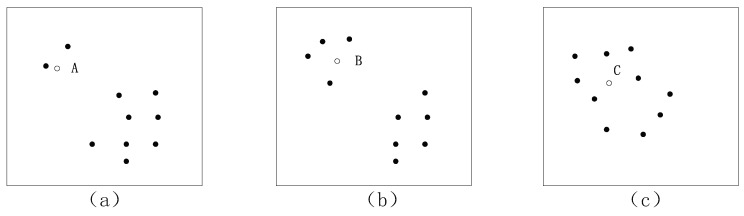
Data distribution scatterplot for different situations.

As an important parameter, the choice of *k* is an open question, and it is usually critical in the k-NN data reconstruction method. Smaller values of *k* cannot reflect the global data features accurately; larger values of *k* reduce the effect of noise but make boundaries between close and long-range samples less distinct. So, a practical approach is to try several different values of *k* using historical data and choose the one that gives the best cross-validation result. In the k-NN reconstruction method, the influence of different *k* values on the reconstruction accuracy can vary in magnitude. In other words, it can determine the accuracy of the fault diagnosis. In order to better introduce the reconstruction methods, three scatterplots are given in Figure 3, where A, B, and C are samples which need to be reconstructed.

Through the summary and comparison using the above methods in Section 4.1 and Section 4.2, we can appreciate that different *k* values are suitable for different situations. With the data distribution shown in Figure 3a, if data point A needs to be reconstructed, k=2 can be applied, and the reconstruction result is accurate because there are a few neighboring samples for sample A on the basis of experience. The result is more suitable for data point B in Figure 3b when k=4, and a reasonable *k* can be determined according to Equation (16). The CNN method has reasonable computing processes for parameter *k*; it avoids the imprecise selection of parameter *k*, so this method can be used as a common approach. So, in Figure 3a, the reconstruction accuracy is satisfactory when k=2; k=4 is suitable for Figure 3b; for Figure 3c, *k* is an open question that needs to be further assessed according to Equation (16).

### 4.4. Faulty Variable Identification Method

When the fault has been detected, the next goal is to identify the faulty variables: the k-NN variable contribution method is used in this paper. This method can be used as a guide to reconstruct the variables.

**Figure 4:**
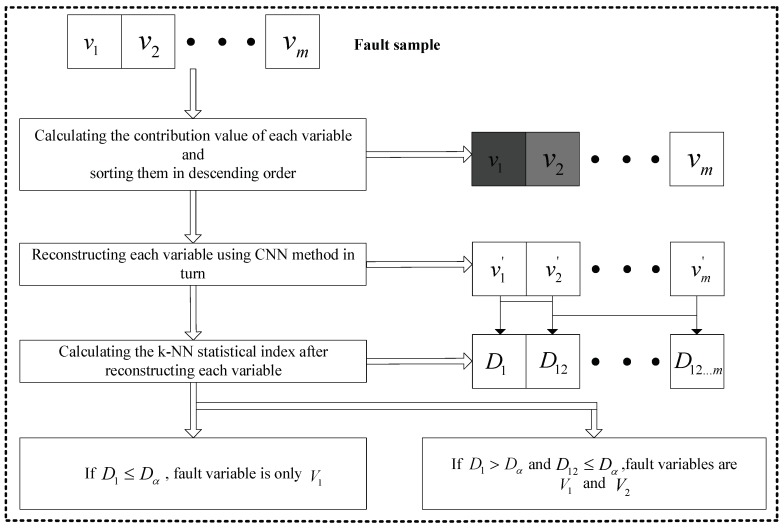
Flowchart of faulty variable identification.

Suppose that there is a sample $\left(v_{1},v_{2},\ldots ,v_{m}\right)$ which contains a fault. The steps of faulty variable identification on the basis of reconstruction are as follows:

Step 1: Calculate the contribution values of each variable and sort them in descending order. The deeper the color, the bigger the contribution value in Figure 4;

Step 2: Reconstruct each variable using the CNN method in turn;

Step 3: Calculate the k-NN statistical index after reconstructing each variable. $D_{1}$ represents the statistical index after reconstructing $v_{1}$, $D_{12}$ is the statistical index after reconstructing $v_{1}$ and $v_{2}$;

Step 4: For example, if $D_{1}\;$<$\;D_{\alpha }$, the faulty variable is only $v_{1}$; if $D_{1}\;$>$\;D_{\alpha }$ and $D_{12}\;$<$\;D_{\alpha }$, the faulty variables are $v_{1}$ and $v_{2}$.

## 5. Illustrative Example

In this section, two examples are introduced to illustrate the performance of the proposed method. In Section 5.1, a simulated numerical example mainly focuses on validating the performances of the CNN data reconstruction strategy and multi-sensor faulty variable identification. The reconstruction results of the CNN and k-NN methods are also given and compared in this simulation. In addition, as a practical industrial example, the Continuous Stirred Tank Reactor system is used to verify the validity of the proposed algorithm in Section 5.2.

### 5.1. An Illustrative Numerical Example

To confirm the specific test purpose, a numerical simulation was designed and constructed in this work. The numerical simulation included seven variables which were driven by two latent variables, $s_{a}$ and $s_{b}$. The simulation data can be generated from the system of equations as follows.

(17) $$\left\{\begin{array}{c} v_{1}=0.3217s_{a}+0.4821s_{b}+e_{1},\\ v_{2}=0.2468s_{a}+0.1766s_{b}+e_{2},\\ v_{3}=0.8291s_{a}+0.4009s_{b}^{2}+e_{3},\\ v_{4}=0.7382s_{a}^{3}+0.0566s_{b}+e_{4},\\ v_{5}=0.3972s_{a}^{2}+0.8045s_{b}^{3}+e_{5},\\ v_{6}=0.6519s_{a}^{2}s_{b}+0.2071s_{b}+e_{6},\\ v_{7}=0.4817s_{a}+0.4508s_{a}s_{b}+e_{7}. \end{array}\right.$$

where $e_{1}$–$e_{7}$ are zero-mean white noises with a standard deviation of 0.01. The changes in the two data sources $s_{a}$ and $s_{b}$ are used to reflect shifts in operating conditions.

(18) $$\left\{\begin{array}{c} s_{a}:uniform\left(-10,7\right),\\ s_{b}:N\left(-15,1\right). \end{array}\right.$$

First, a total of 500 samples were generated as the training data set according to Equations (17) and (18). To test the performance of CNN data reconstruction and faulty variable identification, two test data sets were generated which each contained 500 samples: test data 1 are normal (assuming $v_{1}$ is missing from 151 to 175) and test data 2 are faulty. The fault is added in the following way. Fault case: the system initially runs under normal operating conditions, and a step change is added to $v_{1}$ starting from sample 101 to 150, and the same is done to $v_{2}$ and $v_{7}$ starting from sample 401 to 450. The fault amplitudes are 10%, 8%, and 15%, respectively.

**Figure 5:**
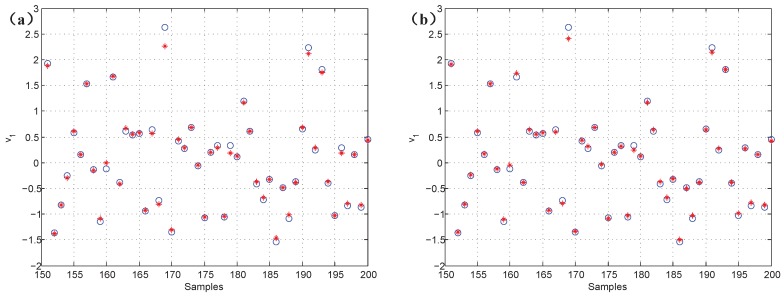
The comparison of reconstructed data and original data; “*” represents reconstructed data, and “o” represents original data. (**a**) k-NN; (**b**) CNN.

To verify the validity of the proposed CNN data reconstruction methods, Figure 5 gives the reconstruction results of the missing values and the original data. In Figure 5a,b, the results show that the k-NN and CNN data reconstruction methods have some effect. However, the accuracy of the CNN method gradually improves. The analysis reveals that the average errors are 3.88% and 3.15% between the reconstructed data and original data, respectively. The slight error demonstrates the effectiveness of the proposed method.

**Figure 6:**
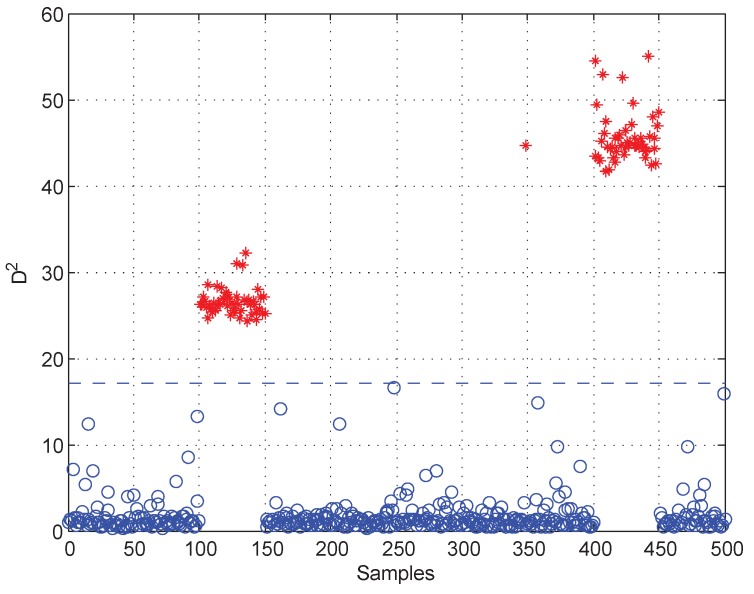
Fault detection result of test data 2.

**Figure 7:**
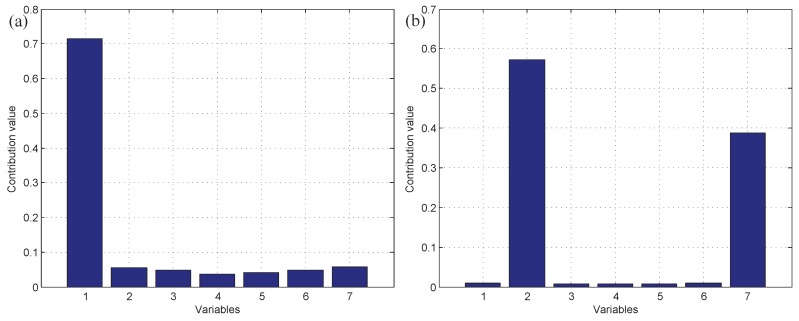
The variable contribution plots of the k-NN method. (**a**) Sample 121; (**b**) sample 421.

Moreover, test data 2 were applied for faulty variable identification. First, the fault detection process should be implemented to verify whether there is a fault. Figure 6 gives the fault detection result of test data 2, and it reveals that faults occur in samples 101–150 and 401–450. Second, the k-NN variable contribution method was used as a guide to reconstruct the variables. Figure 7a,b give the variable contribution plots of the k-NN method in samples 121 and 421. In Figure 7a, $v_{1}$ has the greatest contribution value, which indicates that $v_{1}$ may be the faulty variable in sample 121. Similarly, $v_{2}$ and $v_{7}$ may be the faulty variables in sample 421.

**Figure 8:**
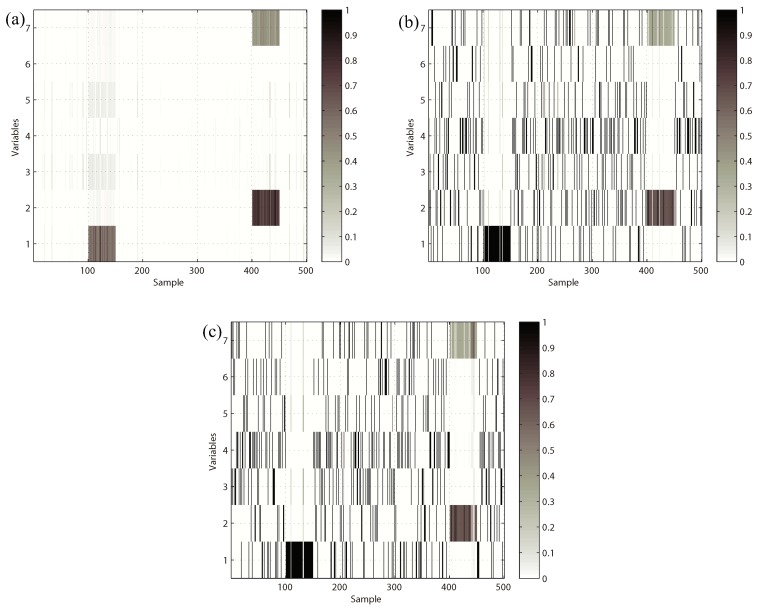
Variable contribution plots. (**a**) k-NN variable contribution, (**b**) PCA-SPE contribution plot, (**c**) RBC method.

In order to compare the proposed method with the traditional methods, the results of anomalous variable recognition of the three different methods are given in Figure 8. Figure 8a is the result of the k-NN variable contribution method. Figure 8b,c are the recognition results of the PCA-SPE-based contribution plot and RBC-based contribution plot, respectively. The darker the color in the graph, the greater the contribution value of the variable. It can be seen that the PCA and RBC methods can show the contribution value of abnormal variables in the fault period, but they cannot avoid the diffusion effect caused by PCA data transformation. In contrast, Figure 8a has a better effect, and the diffusion effect between variables is eliminated obviously, which verifies the effectiveness of the proposed method.

**Figure 9:**
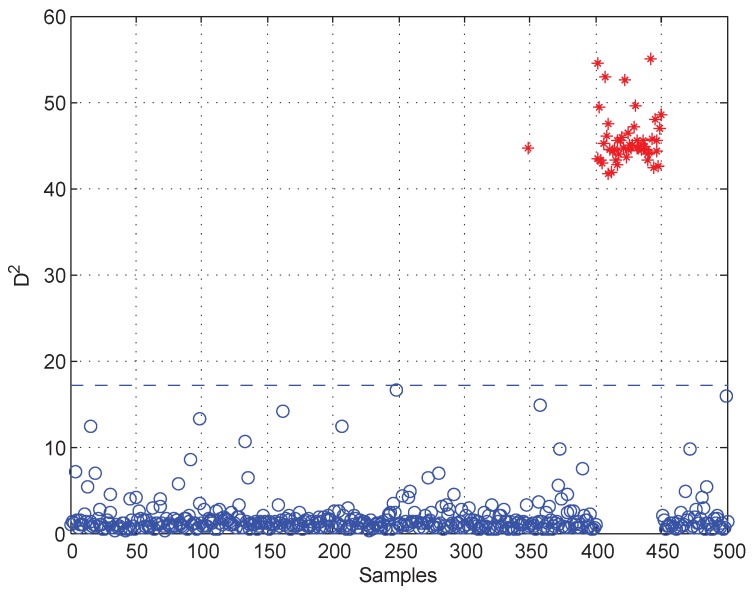
Fault detection result after reconstructing $v_{1}$.

**Figure 10:**
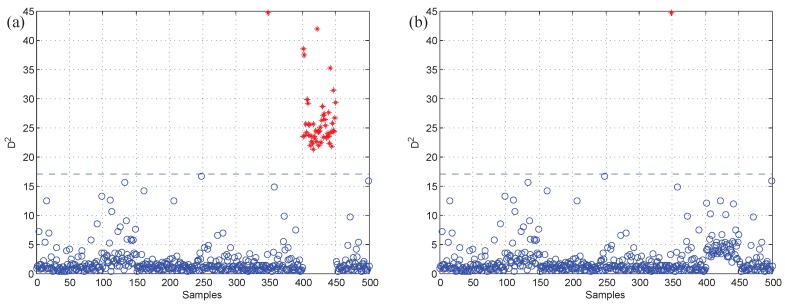
Fault detection results (**a**) after reconstructing $v_{2}$, (**b**) after reconstructing $v_{2}$ and $v_{7}$.

After the guidance provided by the variable contribution plots, the CNN reconstruction method was applied to reconstruct each variable in turn. Between samples 101 and 150, $v_{1}$ should be reconstructed first, and Figure 9 shows the detection result of doing so. We can observe that there are no faults when $v_{1}$ has been reconstructed, so the faulty variable $v_{1}$ is between samples 101 and 150. In the same way, $v_{2}$ and $v_{7}$ were reconstructed one after another. The fault detection results are shown in Figure 10. In Figure 10a, the statistical index decreases after reconstructing $v_{2}$, but it is still beyond the control limit for samples 401–450. Figure 10b reveals that there were no faults when $v_{7}$ was reconstructed. Therefore, the faulty variable is $v_{1}$ from samples 101 to 150, and $v_{2}$ and $v_{7}$ are faulty variables from samples 401 to 450. Table 1 records the “fault–symptom” relationship in this case and accurately shows the variables that have abnormal events in different periods of time. The result of recognition is consistent with the result of the failure setting.

### 5.2. Case Study of Continuous Stirred Tank Reactor System

**Figure 11:**
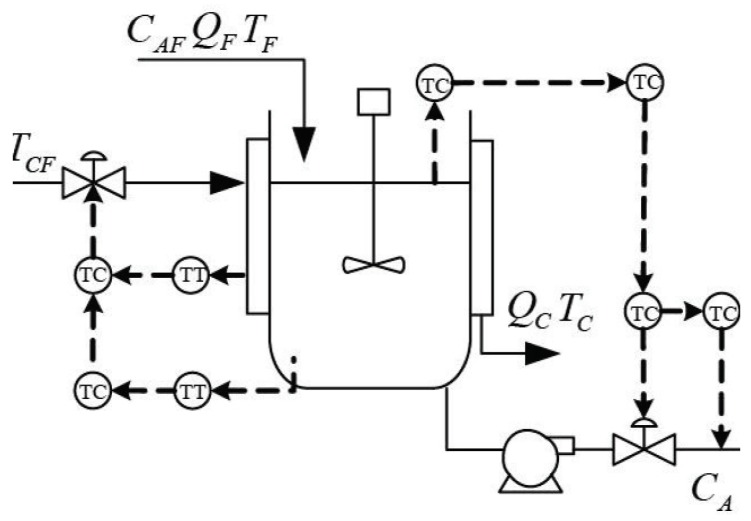
Diagram of the CSTR process.

In the previous subsection, the proposed method was illustrated by a numerical example. However, the correlative influences among variables are weak in this process, and only the key variables that influence the fault can be recognized. In this subsection, the proposed faulty variable identification method is reported according to the Continuous Stirred Tank Reactor (CSTR) system test [36,37], as shown in Figure 11. The data of normal and faulty conditions are generated separately in this process. There are ten process variables (see Table 2), and Gaussian noises are added to all measurements. The simulation generates normal operating data and six kinds of fault pattern data (see Table 3). These faults contain operating condition change, process parameter change, and sensor bias. During the process simulation, 600 normal samples were stored as training data; the fault was introduced after the 300th sample for each fault pattern data.

**Figure 12:**
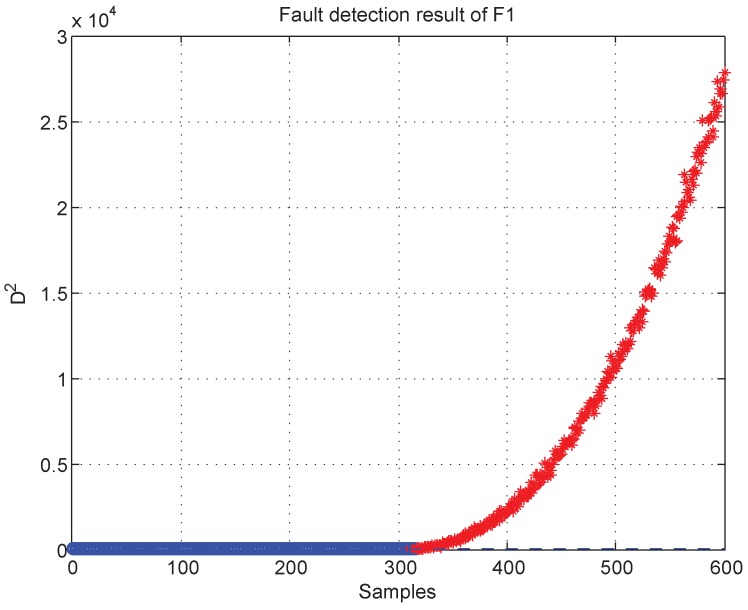
Fault detection result of $F_{1}$.

**Figure 13:**
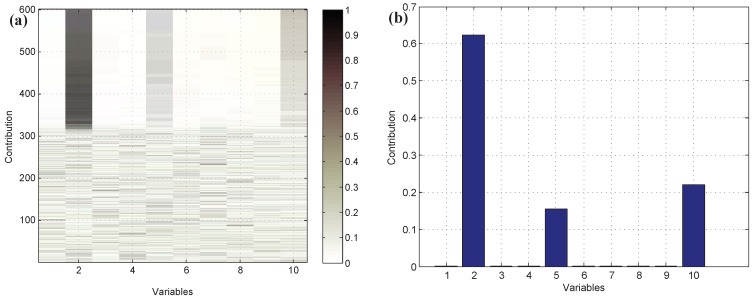
The contribution values of all variables: (**a**) all of the samples, (**b**) the 521th sample.

**Figure 14:**
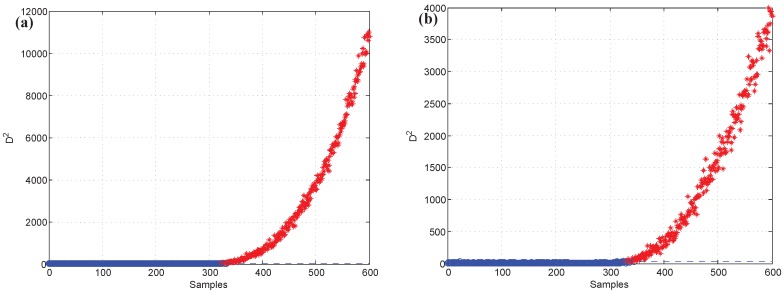

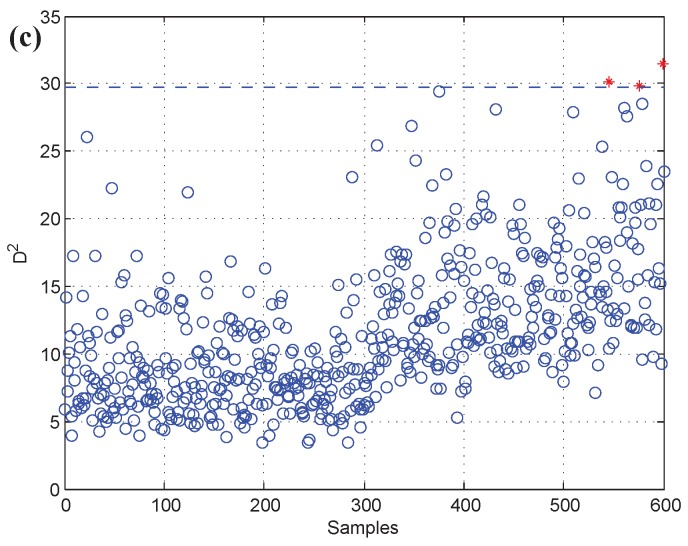
Fault detection result (**a**) after reconstructing variable 2, (**b**) after reconstructing variables 2 and 10, (**c**) after reconstructing variables 2, 10, and 5.

The k-NN fault detection model was constructed to monitor the real-time process, and $F_{1}$ (the coolant feed temperature ramps down) was simulated to implement the identification of faulty variables. First, the k-NN monitoring results of $F_{1}$ are shown in Figure 12, which reveals that the fault was introduced after the 300th sample. Second, once an abnormality was found, the CNN reconstruction method was applied to reconstruct and identify the faulty variables. Figure 13a,b give the contribution values of all variables to the control index. Figure 13a shows that variables 2, 10, and 5 (concentration of species A in feed stream, coolant flow rate, and temperature of coolant in the cooling jacket) have larger contribution values after the 300th sample. Figure 13b gives the contribution values of all variables for sample 521. The detection results after reconstructing the variables that have greater contribution values are shown in Figure 14. Figure 14a is the fault detection result after reconstructing variable 2; the statistical index is reduced compared with Figure 12. When variable 10 is also reconstructed, the statistical index continues to decrease, as shown in Figure 14a,b. Figure 14c gives the fault detection result after reconstructing variable 5, and the statistical index is in control at the moment; this means that there is no exception. So, the faulty variables are 2, 10, and 5. Table 4 records the “fault–symptom” relationship of this case and accurately shows the variables that have abnormal events at different periods of time. This case study validates the effectiveness of the proposed method for faulty variable recognition, especially for the situation of multiple variable faults.

## 6. Discussion and Conclusions

In this work, a novel fault identification method for chemical processes based on k-NN variable contribution and CNN data reconstruction methods was presented, and the results showed that it can reveal all faulty variables accurately. This method has a certain generalizability and can be applied to different chemical processes. This paper first reviewed the k-NN fault detection method for process monitoring. Then, a contribution plot method based on k-NN was proposed for calculating the contribution index of each variable. The feasibility of the k-NN variable contribution method was analyzed in two cases: a single abnormal variable and multiple abnormal variables. This method uses k-NN variable contribution theory to evaluate which variables are most likely to be abnormal. Finally, the fault variable identification method based on the CNN data reconstruction strategy was presented and applied to restructure the variables which have the larger contribution. When there are no abnormalities in the processes, the restructured variables are the faulty variables. The reliability and validity of the proposed method were verified by a numerical example and the Continuous Stirred Tank Reactor system. In Section 5.1, the simulated numerical example mainly focused on validating the performances of the CNN data reconstruction strategy and multi-sensor faulty variable identification. The reconstruction results of the CNN and k-NN methods were also given and compared. The simulation results show the “fault–symptom” relationship for different periods of time. The result of recognition is consistent with the result of the failure setting. In addition, as a practical industrial example, the Continuous Stirred Tank Reactor system was used to verify the validity of the proposed algorithm in Section 5.2.

The proposed method has the following advantages: (1) the k-NN method can deal with non-Gaussian and nonlinear characteristics of modeling data effectively for fault detection; (2) the CNN method is far superior for data reconstruction, and it can guarantee the accuracy of fault recognition. However, it is worth noting that the selection of parameter *k* is still an open question in the k-NN method. More advanced parameter optimization methods may yield a more accurate result. So, we will consider the weight as a new idea and apply the weighted method to reconstruct variable data, which may have a better result than the method discussed in this work. In addition, this paper introduces the proposed method in detail and compares it with the traditional methods (PCA-SPE-based contribution plot and RBC-based contribution plot), the validity of the proposed algorithm is verified, but we will also consider analysing the experimental results using some superior statistical testing methods [38,39] for our future works.

## Figures and Tables

**Table 1:** Results of Abnormal Variable Recognition.

Samples	Abnormal Variables
101–150	$v_{1}$
401–450	$v_{2}$, $v_{7}$

**Table 2:** Monitoring Variable in the CSTR Process.

No. Variable	Measured Variable	Meas. Variables
1	$C_{A}$	concentration of species A in the reactor
2	$C_{AF}$	concentration of species A in feed stream
3	*T*	reactor temperature
4	$T_{F}$	temperature of feed stream
5	$T_{C}$	temperature of coolant in the cooling jacket
6	$T_{CF}$	temperature of coolant feed
7	*h*	liquid level of reactor
8	$Q_{O}$	outlet flow rate
9	$Q_{F}$	feed flow rate to the reactor
10	$Q_{C}$	coolant flow rate

**Table 3:** Fault Description of the CSTR Process.

No. Variable	Measured Variable
$F_{1}$	the coolant feed temperature ramps down
$F_{2}$	the feed concentration ramps up
$F_{3}$	the feed temperature ramps up
$F_{4}$	the heat transfer coefficient ramps down
$F_{5}$	catalyst deactivation
$F_{6}$	the coolant temperature measurement has a bias

**Table 4:** Results of Abnormal Variable Recognition.

Samples	Abnormal Variables
111–115	$v_{2}$
116–200	$v_{2}$, $v_{5}$, $v_{10}$

## References

[1] Ge Z., Song Z., Gao F. (2013). Review of Recent Research on Data-Based Process Monitoring. Ind. Eng. Chem. Res..

[2] Kano M., Nakagawa Y. (2008). Data-based process monitoring, process control, and quality improvement: Recent developments and applications in steel industry. Comput. Chem. Eng..

[3] Cai J., Ferdowsi H., Sarangapani J. (2016). Model-based fault detection, estimation, and prediction for a class of linear distributed parameter systems. Automatica.

[4] Zhang X., Kano M., Li Y. (2018). Principal polynomial analysis for fault detection and diagnosis of industrial processes. IEEE Access.

[5] Pan Y., Yang C., An R. (2017). Robust principal component pursuit for fault detection in a blast furnace process. Ind. Eng. Chem. Res..

[6] Funa Z., Ju P., Chenglin W. (2018). Average Accumulative Based Time Variant Model for Early Diagnosis and Prognosis of Slowly Varying Faults. Sensors.

[7] Ge Z., Song Z., Ding S., Huang B. (2017). Data mining and analytics in the process industry: The role of machine learning. IEEE Access.

[8] Zhang R., Peng Z., Wu L., Yao B., Guan Y. (2017). Fault Diagnosis from Raw Sensor Data Using Deep Neural Networks Considering Temporal Coherence. Sensors.

[9] Freeman J. (1992). A User’s Guide to Principal Components. J. Oper. Res. Soc..

[10] Wang J., He Q. (2010). Multivariate Statistical Process Monitoring Based on Statistics Pattern Analysis. Ind. Eng. Chem. Res..

[11] Yuan Y., Chen T., Gao F. (2010). Multivariate statistical monitoring of two-dimensional dynamic batch processes utilizing non-Gaussian information. J. Process Control.

[12] Qin S. (2010). Statistical process monitoring: Basics and beyond. J. Chemom..

[13] Li Y., Zhang X. (2015). Variable moving windows based non-Gaussian dissimilarity analysis technique for batch processes fault detection and diagnosis. Can. J. Chem. Eng..

[14] Chiang L., Russell E., Braatz R. (2000). Fault diagnosis in chemical processes using Fisher discriminant analysis, discriminant partial least squares, and principal component analysis. Chemom. Intell. Lab. Syst..

[15] Chiang L., Braatz R. (2003). Process monitoring using causal map and multivariate statistics: Fault detection and identification. Chemom. Intell. Lab. Syst..

[16] Nomikos P., Macgregor J. (2010). Monitoring batch processes using multiway principal component analysis. AIChE J..

[17] Zhang N., Wu L., Yang J., Guan Y. (2018). Naive Bayes Bearing Fault Diagnosis Based on Enhanced Independence of Data. Sensors.

[18] Youssef T., Chadli M., Karimi H.R., Wang R. (2017). Actuator and sensor faults estimation based on proportional integral observer for TS fuzzy model. J. Frankl. Inst..

[19] Zhang X., Li Y., Kano M. (2015). Quality Prediction in Complex Batch Processes with Just-in-Time Learning Model Based on Non-Gaussian Dissimilarity Measure. Ind. Eng. Chem. Res..

[20] Wang G., Liu J., Li Y. (2015). Fault Detection Based on Diffusion Maps and k Nearest Neighbor Diffusion Distance of Feature Space. J. Chem. Eng. Jpn..

[21] Zhao S., Zhang J., Xu Y. (2004). Monitoring of Processes with Multiple Operating Modes through Multiple Principle Component Analysis Models. Ind. Eng. Chem. Res..

[22] Zhao S., Zhang J., Xu Y. (2006). Performance monitoring of processes with multiple operating modes through multiple PLS models. J. Process Control.

[23] Yoo C., Villez K., Lee I. (2007). Multi-model statistical process monitoring and diagnosis of a sequencing batch reactor. Biotechnol. Bioeng..

[24] Duda R., Hart P., Stork D. (2001). Pattern Classification.

[25] He Q., Wang J. Principal component based k-nearest neighbor rule for semiconductor process fault detection. Proceedings of the 2008 American Control Conference.

[26] Guo X., Yuan J., Li Y. (2014). Feature space k nearest neighbor based batch process monitoring. Acta Autom. Sin..

[27] Westerhuis J., Gurden S., Smilde A. (2000). Generalized contribution plots in multivariate statistical processmonitoring. Chemom. Intell. Lab. Syst..

[28] Yang Y. (2002). Multivariate Statistical Process Monitoring and Fault Diagnosis Method and Its Application. Ph.D. Thesis.

[29] Kourti T., Macgregor J. (1996). Multivariate SPC Methods for Process and Product Monitoring. J. Qual. Technol..

[30] Dunia R., Qin S. (1998). Subspace approach to multidimensional fault identification and reconstruction. AIChE J..

[31] Alcala C., Qin S. (2009). Reconstruction-based contribution for process monitoring. Automatica.

[32] Yue H., Qin S. (2001). Reconstruction-Based Fault Identification Using a Combined Index. Ind. Eng. Chem. Res..

[33] Liu J. (2012). Fault diagnosis using contribution plots without smearing effect on non-faulty variables. J. Process Control.

[34] Chen Q., Wynne R., Goulding P. (2000). The application of principal component analysis and kernel density estimation to enhance process monitoring. Control Eng. Pract..

[35] Wang G., Liu J., Li Y. (2015). Fault diagnosis using kNN reconstruction on MRI variables. J. Chemom..

[36] Shang L., Liu J., Zhang Y. (2016). Efficient recursive canonical variate analysis approach for monitoring time-varying processes. J. Chemom..

[37] Shang L., Liu J., Zhang Y. (2016). Recursive Fault Detection and Identification for Time-Varying Processes. Ind. Eng. Chem. Res..

[38] Derrac J., García S., Molina D., Herrera F. (2011). A practical tutorial on the use of nonparametric statistical tests as a methodology for comparing evolutionary and swarm intelligence algorithms. Swarm Evol. Comput..

[39] Fan G., Peng L., Hong W. (2018). Short term load forecasting based on phase space reconstruction algorithm and bi-square kernel regression model. Appl. Energy.

